# Anti-inflammatory and analgesic activities of solvent fractions of the leaves of *Moringa stenopetala* Bak. (Moringaceae) in mice models

**DOI:** 10.1186/s12906-017-1982-y

**Published:** 2017-09-29

**Authors:** Yohannes Tamrat, Teshome Nedi, Solomon Assefa, Tilahun Teklehaymanot, Workineh Shibeshi

**Affiliations:** 1 Department of pharmacology and clinical pharmacy, School of Pharmacy, College of Health Sciences, Addis Ababa University, Addis Ababa, Ethiopia; 2 Aklilu Lemma institute of pathobiology, Addis Ababa University, Addis Ababa, Ethiopia

**Keywords:** Analgesic activity, Anti-inflammatory activity, Radiant tail-flick latency, Acetic acid induced writhing, Carrageenan induced paw edema, *Moringa stenopetala*

## Abstract

**Background:**

Many people still experience pain and inflammation regardless of the available drugs for treatments. In addition, the available drugs have many side effects, which necessitated a quest for new drugs from several sources in which medicinal plants are the major one. This study evaluated the analgesic and anti- inflammatory activity of the solvent fractions of *Moringa stenopetala* in rodent models of pain and inflammation.

**Methods:**

Successive soxhlet and maceration were used as methods of extractions using solvents of increasing polarity; chloroform, methanol and water. Swiss albino mice models were used in radiant tail flick latency, acetic acid induced writhing and carrageenan induced paw edema to assess the analgesic and anti-inflammatory activities. The test groups received different doses (100 mg/kg, 200 mg/kg and 400 mg/kg) of the three fractions (chloroform, methanol and aqueous). The positive control groups received morphine (20 mg/kg) or aspirin (100 mg/kg or 150 mg/kg) based on the respective models. The negative control groups received the 10 ml/kg of vehicles (distilled water or 2% Tween 80).

**Results:**

In all models, the chloroform fraction had protections only at a dose of 400 mg/kg. However, the methanol and aqueous fraction at all doses have shown significant central and peripheral analgesic activities with a comparable result to the standards. The aqueous and methanol fractions significantly reduced carrageenan induced inflammation in a dose dependent manner, in which the highest reduction of inflammation was observed in aqueous fraction at 400 mg/kg.

**Conclusion:**

This study provided evidence on the traditionally claimed uses of the plant in pain and inflammatory diseases, and *Moringa stenopetala* could be potential source for development of new analgesic and anti-inflammatory drugs.

## Background

Pain is always a subjective an unpleasant sensory and emotional experience associated with actual or potential tissue damage and described in terms of such damage [1]. There may be a strong emotional component contributing to the pain experience, but that does not mean that the suffering is less important [2]. It is the most common reason a patient sees a physician. For most patients, it is of short duration and quickly forgotten [3]. When chronic, it markedly decreases individuals’ health status and quality of life and can detrimentally affect the families of patients. It often interferes with every day work activities [4].Unrelieved acute pain can cause chronic pain, and long standing pain can cause anatomical and even genetic changes in the nervous system [2].

Inflammation on the other hand is a physiological response of living tissues to injury [5]. Although the inflammatory response is essential for host defense, it is very much a double-edged sword which can lead to an organ failure and/or death [6]. To relieve the damage they cause and to reduce their effect in quality of life, it might be necessary to take pharmacological agents against pain and inflammation. Non-steroidal anti-inflammatory drugs (NSAIDs), corticosteroids, and opiates have been used classically in these conditions [7, 8]. However, due to extensive use of analgesic and anti-inflammatory agents, the toxicity and untoward effects occur many times, especially when therapy of pain and inflammation involves use of higher doses for longer periods [9]. Gastrointestinal disturbances, respiratory depression, possible dependence [7], constipation [10], renal dysfunction [11], peptic ulcer and bleeding [12] are some of the commonly encountered untoward effects of analgesic and anti-inflammatory agents.

Natural products derived from medicinal plants are becoming preferred alternative remedies. By screening medicinal plants with acclaimed analgesic and anti-inflammatory use, safe and effective analgesic and anti-inflammatory drugs might be discovered [13]. It is therefore essential that efforts should be made to introduce new compounds derived from medicinal plants to the drug arsenal against pain and inflammation [14].

There are 6500 species of higher plants in Ethiopia making the country one of the most diverse floristic regions in the world [15]. It is estimated that about 80% of the population use plant based traditional medicine as their major primary health care system [16]. Many plants are used as analgesic and/or anti-inflammatory agents in traditional medicine practice of Ethiopia. Some of these plants include: *Allium sativum* [17], *Zingiber officinale* [18], *Nigella sativum* [18], *Albuca abyssinica* [16], *Ruta chalepensis* [19], and *Moringa stenopetala* [20]*. M. stenopetala* is a native tree in arid and semi-arid regions in the southern Rift Valley of Ethiopia [21]. It is also reported to occur in Djibouti, Uganda and Sudan [22]. It is known by different vernacular names such as “Shiferaw” (Amharic), “Halako” (Gamo & Wollayita) [23], “Shelchada” (Konso), and “Cabbage tree” (English) [24]. Traditionally, the leaves boiled in water, can treat and cure headache, malaria, hypertension and stomach pain [20]. Recently, the in-vivo analgesic and anti-inflammatory activity of the crude leaf extract of the plant has been confirmed by Geremew et al. [25]. This study aimed to further evaluate the analgesic and anti-inflammatory activities of the solvent fractions of *M. stenopetala* in Swiss albino mice models using radiant tail flick latency, acetic acid induced writhing and carrageenan induced paw edema. Radiant tail flick latency, and acetic acid induced writhing which are proven methods to test central and peripheral analgesic activity respectively, while carrageenan induced paw edema model is suitable for testing acute inflammatory responses. In addition to their cost effectiveness, Rodent models of pain have played a dominant role in the study of pain mechanisms [26].

## Methods

### Drugs and chemicals

Analytical grade chemicals and drugs used in study were; aspirin and morphine (EPHARM, Ethiopia), Tween 80% (Uni-chem Chemical Reagents, India), carrageenan (Sigma Aldrich, Germany), normal saline (Fresenius Kabi, India), glacial acetic acid (Carlo erba group reagents, Italy), chloroform and methanol (Carlo erba group reagents, Italy).

### Plant material collection and extract preparation

The fresh leaves of *M. stenopetala* were collected in February 2015, from Wolaita sodo town, 313 km south of Addis Ababa. Taxonomic identifications were then established (voucher sample no. MS001) at the Department of Biology, National Herbarium, Addis Ababa University. The collected leaves of *M. stenopetala* were thoroughly washed with distilled water to remove dirt and soil. The leaves were air dried under shade and then pulverized to a coarse powder. Successive soxhlet and maceration techniques were used for the extraction of plant material. The powdered leaves were placed in the extraction chamber of the soxhlet apparatus. For each 50 g of plant powder, 300 ml of solvent was used. The leaf powder was subjected to successive soxhlet extraction with two solvents of different polarity (chloroform and absolute methanol).

The first extracting solvent (chloroform) in the flask was heated until clear liquid contents of the chamber siphoned into the solvent flask. The solvents was later removed using rotary evaporator (Buchi Rota vapor, Switzerland) under reduced pressure set at 40 °C followed by the oven at room temperature. And then it was extracted using absolute methanol following the same procedure. Then, the marc of absolute methanol fraction was collected and dried at room temperature to remove the methanol. Finally, the dried marc left from the two solvent extraction was cold macerated in an Erlenmeyer flask with distilled water and allowed to stand at room temperature for a period of 72 h with occasional shaking using mini orbital shaker (Stuart, United Kingdom). It was then filtered with gauze followed by filter paper (Whatman No.1). The residue was re-macerated twice using the same solvent to exhaustively extract the plant material. The filtrate was freeze dried in a lyophilizer (Operon, Korea vacuum limited, Korea) to remove water. After drying, percentage yield of all fractions was determined and it was found to be 4.5%, 7.8% and 6.4%, for chloroform (CF), methanol(MF) and aqueous fractions(AF), respectively. The CF and MF were reconstituted in 2% Tween 80, while the AF was reconstituted in distilled water before administration.

### Animals

Healthy Swiss albino mice(25-35 g), which areaged 6–8 weeks obtained from the animal house of Ethiopian Public Health Institute (EPHI) and from the animal house of School of Pharmacy, Addis Ababa University, were used. Mice were kept in polypropylene cages and maintained at room temperature and on a 12/12 h light-dark cycle with access to standard laboratory pellet food and water ad libitum. They were acclimatized for a week before the commencement of the experiment. All studies were conducted in accordance with international guidelines [27], and approval was assured by ethical review board of School of Pharmacy, Addis Ababa University.

### Preliminary phytochemical screening

Standard phytochemical screening tests were carried out on each of the solvent fractions of *M. stenopetala* for the presence or absence of secondary metabolites using standard procedures [28, 29].

### Acute toxicity test

Acute toxicity test for the leaf fractions of *M. stenopetala* was carried out based on the limit test recommendations of OECD 425 guideline [30], on female mice. Three female Swiss albino mice were used as sighting study for each solvent fraction and fasted for 4 h prior to the experiment and 2 h after the experiment. The mice were administered with a single dose (2000 mg/kg) of CF, MF and AF orally using oral gavage. The mice were then observed for physical or behavioral changes within 24 h strictly, with special attention during the first 4 h. Since no death was observed within 24 h, additional four mice were administered with the same dose of fractions followed by similar strict observation. The observation was done for 4 h with 30 min interval during the experiment and then for 14 consecutive days with an interval of 24 h for the general signs and symptoms of toxicity, food and water intake and mortality.

### Animal grouping and dosing

**Fig. 1:**
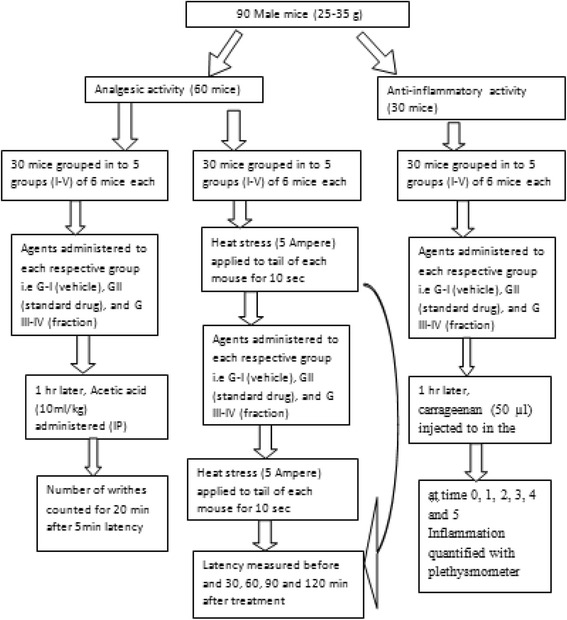
Schematic presentation of the designed experiment

In all models as shown in Fig. 1, male mice were randomly divided into five groups (negative control, positive control and three test groups) comprising of six animals each to perform the analgesic and anti-inflammatory activity test. The first group was assigned as a control and received the vehicle (2% Tween 80 or distilled water) at a volume of 10 ml/kg. The second group was assigned as a positive control and administered with standard drugs (morphine) 20 mg/kg for a radiant tail-flick method. On the other hand, 150 mg/kg dose of Aspirin and 100 mg/kg was administered for acetic acid induced writhing and carrageenan induced paw edema model, respectively. The rest three groups were given different doses (100 mg/kg, 200 mg/kg and 400 mg/kg) of the three different fractions. The chloroform and absolute methanol fractions were reconstituted in 2% Tween 80, while the aqueous fraction was reconstituted in distilled water. Dose selection was made based on OECD [30] guideline after the acute toxicity evaluation of the plant. All administrations were carried orally using an oral gavage.

### Analgesic activity

#### Radiant heat tail-flick method

The radiant heat tail-flick test, originally described by D’Amour and Smith [31] with slight modification [32] was performed to study the analgesic activity of *M. stenopetala*. Before (pretreatment latency) and following administration of the agents as per their grouping, heat stress was applied to tails of mice and the subsequent change in sensitivity was measured using Analgesiometer (Techno Type: Mark-IB, SL.No; 720,121) by maintaining the current intensity passing through the naked nichrome wire at 5 A. The distance between the heat source and the tail skin was 1.5 cm. for the purpose of preventing tissue damage, the cut-off time was set to be 10 s. The time taken by mice to withdraw (flick) the tail was taken as the reaction time. Hence, observations were made at initial reading prior and at an interval of 30, 60, 90 and 120 min after drug administration. Anti-nociception in tail-flick was quantified as the maximum possible effect (MPE) using the following formula [33]:

$$ \mathrm{MPE}\%=\kern0.5em \frac{\mathrm{Post}\kern0.5em \mathrm{treatment}\kern0.5em \mathrm{latency}\hbox{-} \mathrm{Pre}\kern0.5em \mathrm{treatment}\kern0.5em \mathrm{latency}}{\mathrm{Cut}\kern0.5em \mathrm{off}\kern0.5em \mathrm{time}\kern0.5em (10)\hbox{-} \mathrm{Pre}\mathrm{treatment}\kern0.5em \mathrm{latency}}\times 100 $$

### Acetic acid- induced writhing method

The method described by Koster et al. [34] for acetic acid-induced writhing test has been used. Accordingly, overnight fasted mice were grouped into two control and three experimental groups of six mice each and administered with respective doses of solvent fractions and aspirin. To assess analgesic activity of various groups, the number of writhes that was indicated by stretching of the abdomen with simultaneous stretching of at least one hind limb was counted for each mouse for 20 min using a latency period of 5 min, and the percentage was calculated using the formula described below [35].

$$ \%\kern0.5em \mathrm{inhibtion}=\frac{\mathrm{Mean}\kern0.5em \mathrm{number}\kern0.5em \mathrm{of}\kern0.5em \mathrm{writhes}\kern0.5em \left(\mathrm{control}\right)\hbox{-} \mathrm{Mean}\kern0.5em \mathrm{number}\kern0.5em \mathrm{of}\kern0.5em \mathrm{writhes}\kern0.5em \left(\mathrm{treated}\right)}{\mathrm{Mean}\kern0.5em \mathrm{number}\kern0.5em \mathrm{of}\kern0.5em \mathrm{writhes}\kern0.5em \left(\mathrm{control}\right)}\times 100 $$

### Anti-inflammatory activity

#### Carrageenan induced mice paw edema method

The anti-inflammatory activity was evaluated using carrageenan induced paw edema in mice according to the method used in Winter et al. [36] with slight modification. Acute inflammation was produced by injection of carrageenan (1% *w*/*v* carrageenan in normal saline, 50 μl) into the plantar surface of the right hind paw of the mice. Initially, the mice were divided into 5 groups. In the their respective groups, the mice were pre-treated with standard drug, the vehicle and solvent fraction 60 min before injection of carrageenan. The acute phase of inflammatory reaction was quantitated in terms of ml i.e., displacement of water by edema using a digital plethysmometer (Ugo Basile Company: Cat No 7140, Italy) at time 0, 1, 2, 3, 4 and 5 h after carrageenan injection. The percent inhibition of edema was calculated in comparison to the animals in the control group using the following formula [37]:

$$ \%\kern0.5em \mathrm{Inhibition}\kern0.5em \mathrm{of}\kern0.5em \mathrm{paw}\kern0.5em \mathrm{edema}=\frac{\left(\mathrm{Vt}\hbox{-} \mathrm{Vo}\right)\mathrm{control}\hbox{-} \left(\mathrm{Vt}\hbox{-} \mathrm{Vo}\right)\mathrm{Treated}}{\left(\mathrm{Vt}\hbox{-} \mathrm{Vo}\right)\mathrm{control}}\times 100 $$

$$ {\displaystyle \begin{array}{l}\mathrm{Where}:\hbox{-} \mathrm{Vt}:\mathrm{is}\  \mathrm{the}\  \mathrm{right}\  \mathrm{hind}\ \mathrm{paw}\ \mathrm{t}\mathrm{hickness}\  \mathrm{volume}\ \left(\mathrm{in}\ \mathrm{ml}\right)\ \mathrm{at}\ \mathrm{t}\mathrm{ime}\ \mathrm{t}\hfill \\ {}\kern6.25em \mathrm{Vo}:\mathrm{is}\  \mathrm{the}\  \mathrm{right}\  \mathrm{hind}\ \mathrm{paw}\ \mathrm{t}\mathrm{hickness}\  \mathrm{volume}\ \left(\mathrm{in}\ \mathrm{ml}\right)\ \mathrm{beforecarrageenan}\  \mathrm{injection}\hfill \end{array}} $$

### Statistical Analysis

All data found from the research were expressed as mean ± standard error of the mean (SEM). Data was analyzed by one way analysis of variance (ANOVA) followed by Tukey post-hoc test to determine statistical significance using statistical package for social science (SPSS). Linear regression was also used where appropriate. *P* values less than 0.05 were taken as statistically significant.

## Results

### Preliminary phytochemical screening

The objective of preliminary phytochemical screening was to explore the types of secondary metabolites in extracts based on qualitative color changes of test reagents which will give a clue to possible mechanisms of analgesic and anti-inflammatory effects. The solvent fractions from leaves of *M. stenopetala* revealed the presence of various secondary metabolites based on preliminary phytochemical screening test. Accordingly, the chloroform fraction contains alkaloids and steroids only. The aqueous fraction contained tannins, flavonoids, saponins and cardiac glycosides. However, methanol fraction was richest containing tannins, alkaloids, saponins, terpenoids, and anthraquinones.

The objectives of acute toxicity study were to know safety margin of extract and select therapeutic dose levels. In the acute toxicity study, none of the animals showed behavioral, neurological or physical changes characterized by symptoms such as reduced motor activity, restlessness, convulsions, coma, diarrhea and lacrimation at the limit dose of 2000 mg/kg of the solvent fractions of *M. stenopetala*. Moreover, no mortality was observed in 24 h as well as in the next 14 days.

### Analgesic activity

#### Radiant tail flick latency

Radiant tail flick test was conducted to assess possible centrally mediated analgesic activity of extracts.

In the radiant heat tail-flick method, the largest dose of CF and all doses of MF had shown a prolonged heat stress tolerance capacity of the mice in comparison with the control (*p* < 0.001) at all-time points (Table 1). The middle dose of CF has exhibited statistically significant (p < 0.001) reduction in pain sensation at 90 and 120 min interval. The standard drug morphine caused a maximum (90.3%) increase in analgesic activity at 90 min. Among the different fraction and doses of the plant, methanol 400 mg/kg showed the highest (46.6%) protection at the 90th min, which is in similar time interval with the peak analgesic effect of morphine. On the other hand, all doses of AF has exhibited a statistically significant (p < 0.001) reduction of pain compared to the control at all-time points (Table 2). The maximum protection was observed at 120 min (44.8%, 70.1% & 73.6% for AF100, AF200 & 400 mg/kg, respectively). The highest and middle doses of the AF shown a comparable effect with morphine (62.1%). Comparing all of the three fractions, the aqueous fraction had shown a better central analgesic activity.

**Table 1:** The effect of chloroform and methanol fractions on radiant tail flick latency and maximum possible protection (%)

	Latency (sec) and Maximum possible protection (%)								
Group	Pretreatment	30 min	%	60 min	%	90 min	%	120 min	%
TE	2.05 ± 0.13	2.12 ± 0.08	–	2.52 ± 0.14	–	2.80 ± 0.16	–	2.66 ± 0.18	–
MO	2.22 ± 0.04	8.25 ± 0.08^a3c3d3h3^	77.5	8.92 ± 0.05^a3c3d3h3^	86.1	9.25 ± 0.04^a3c3d3h3^	90.3	8.56 ± 0.06^a3c3d3h3^	81.5
CF 100	2.15 ± 0.05	2.26 ± 0.06^b3^	1.4	2.54 ± 0.14^b3^	5.0	2.99 ± 0.11^b3^	10.6	2.64 ± 0.1	6.3
CF 200	2.13 ± 0.06	2.35 ± 0.04^b3^	2.8	2.88 ± 0.03^b3^	9.2	3.53 ± 0.09^a3^	17.9	3.88 ± 0.07^h3a3^	22.3
CF 400	2.2 ± 0.04	2.78 ± 0.07^a3^	7.5	3.40 ± 0.09^h2a3^	15.4	5.41 ± 0.09^g2h3^	41.2	4.04 ± 0.07^a3^	23.6
MF 100	2.16 ± 0.05	2.60 ± 0.03^a3^	5.6	3.29 ± 0.06^h3a3^	14.4	3.58 ± 0.09^e3a3^	18.2	4.27 ± 0.07^a3^	27.0
MF 200	2.23 ± 0.05	2.76 ± 0.07^a3^	6.9	3.70 ± 0.08^a3h3^	19.0	4.83 ± 0.07^e2a3^	33.4	4.15 ± 0.07^a3^	24.6
MF 400	2.23 ± 0.06	2.78 ± 0.05^a3^	7.1	3.86 ± 0.04^a3f3g3^	21.0	5.84 ± 0.06^e1a3g3^	46.6	5.41 ± 0.1^e3a3g3^	41.0

Values are expressed as Mean ± S.E.M (*n* = 6); ^a^against control, ^b^against standard drug, ^c^against CF100, ^d^against CF200, ^e^against CF400,^f^against MF 100, ^g^against MF 200, ^h^against MF 400. ^1^ *P* < 0.05, ^2^ *P* < 0.01, ^3^ *P* < 0.001; where *TE* tween eighty, *MO* morphine, CF and MF refers to chloroform and methanol fractions of *Moringa stenopetala* respectively; control received 2% Tween 80 where as standard received Morphine orally; Numbers refer to doses in mg/k

**Table 2:** Effect of aqueous fraction of *Moringa stenopetala* on radiant tail flick latency and maximum possible protection(%)

	Latency (sec) and maximum possible protection (%)								
Groups	0 time	30 min	%	60 min	%	90 min	%	120 min	%
DW	2.18 ± 0.034	2.10 ± 0.1	–	2.18 ± 0.2	–	2.26 ± 0.2	–	2.15 ± 0.1	–
MO	2.23 ± 0.034	9.23 ± 0.09^a3d3^	90.2	8.87 ± 0.21^a3d3^	85.4	7.44 ± 0.09^a3d3^	67.0	7.05 ± 0.06^a3^	62.1
AF 100	2.23 ± 0.052	3.00 ± 0.08^a3b3d1^	10.0	3.67 ± 0.17^a2e3b3^	18.6	4.49 ± 0.19^e3a3d3^	29.2	5.71 ± 0.09^a3b3d3e3^	44.8
AF 200	2.33 ± 0.049	3.18 ± 0.11^a3e3^	11.1	3.87 ± 0.07^a3b3e3^	29.2	5.01 ± 0.11^a3b3e3^	34.9	7.7 ± 0.22^a3c3^	70.1
AF 400	2.13 ± 0.044	3.61 ± 0.1^a3b3c3d3^	18.9	4.79 ± 0.09^a3b3c3d3^	44.8	6.26 ± 0.05^a3c3d3^	70.1	7.71 ± 0.27^a3c3^	73.6

Values are expressed as Mean ± S.E.M (*n* = 6); ^a^against the control, ^b^against the standard drug, ^c^against AF100, ^d^against AF200, ^e^against AF400, ^1^ *P* < 0.05, ^2^ *P* < 0.01, ^3^ *P* < 0.001; AF refers to aqueous fraction of *Moringa stenopetala*, *MO* morphine; and DW stands for Distilled Water. Control received distilled water (10 ml/kg), whereas standard received Morphine (20 mg/kg) orally; Numbers refers to doses in mg/kg

#### Acetic acid induced writhing test

This test was performed to evaluate peripheral analgesic effect of extracts to chemical –induced pain stimulus. The solvent fractions of *M. stenopetala*, acetic acid- induced writhing model of analgesia was used. In this method the number of writhes (in 10 min) was highest in Tween 80 treated control group (57.0 ± 2.89) and lowest in AF 400 group (23.2 ± 0.95) as shown in Tables 3 and 4. Generally, the results of all doses of MF were significant and comparable with the effect of aspirin 150 mg/Kg in analgesic activity, while the CF had shown a protection only at a dose of 400 mg/kg (*p* < 0.01) in comparison with the control (Table 3). The AF at the highest dose (400 mg/kg) had the best protection (58.0%) even better than the standard drug (Table 4).

**Table 3:** Effect of methanolic and chloroform fractions of leaf of *Moringa stenopetala* on acetic acid induced writhing test in mice

Group	No. of writhing	% Inhibition
TE	57.0 ± 2.89	–
ASA 150	28.3 ± 0.88^a3^	50.4
CF 100	54.5 ± 1.18	4.4
CF 200	51.7 ± 0.76	9.3
CF 400	48.5 ± 0.67^a2b3f3^	14.9
MF 100	32.8 ± 0.60^a3h1^	42.5
MF 200	29.8 ± 0.60^a3^	47.7
MF 400	26.5 ± 0.67^a3^	53.5

Values are expressed as Mean ± S.E.M (n = 6);^a^against the control, ^b^against the standard drug, ^f^against MF 100, ^h^against MF 400. ^1^ *P* < 0.05, ^2^ *P* < 0.01, ^3^ *P* < 0.001; *TE* tween 80, *ASA* aspirin, CF and MF refers to chloroform and methanol fractions of M*oringa stenopetala,* respectively. Numbers refer to dose in mg/kg

**Table 4:** Effect of Aqueous leaves fraction of *Moringa stenopetala* on acetic acid induced writhing test in mice

Group	No. of writhing	% Inhibition
DW	55.3 ± 2.47	–
ASA 150	28.3 ± 0.88^a3^	48.8
AF 100	29.3 ± 0.56^a3e1^	47.0
AF 200	26.8 ± 0.70^a3^	51.5
AF 400	23.2 ± 0.95^a3^	58.0

Values are expressed as Mean ± S.E.M (*n* = 6); ^a^against control, ^e^against AF400, ^1^ *P* < 0.05, ^3^ *P* < 0.001; *DW* distilled water, *ASA* aspirin, AF refers to aqueous fraction of *Moringa stenopetala;* Numbers refer to dose in mg/kg

### Anti-inflammatory activity

#### Carrageenan induced paw edema

This test was conducted to assess anti-inflammatory effect of extracts following induction of inflammatory pain by injection of carrageenan. Except at a dose of 200 mg/kg at 3rd hour (*p* < 0.01), a strong inhibition (*p* < 0.001) of the paw edema was observed with the lower, middle and higher doses of MF starting from the second hour after carrageenan injection. Whereas, only the higher dose of CF had shown a significant inhibitory activity against the edema caused by sub-planar injection of carrageenan (*p* < 0.05 at 2nd and 3^rd^hours, *p* < 0.01 at 4th and *p* < 0.001 at 5th hour). As shown in Table 5, the maximum protection from increase in paw volume was observed at the second hour from all doses of CF, MF and the standard drug aspirin, at 400 mg/kg doses, CF and MF showed 24.4%, and 52.2% inhibition at the second hour, respectively. While the protection was 63.9% for aspirin treated group.

**Table 5:** Effects of the chloroform and methanol fractions of *Moringa stenopetala* on carrageenan- induced paw edema and percent inhibition from increase in paw volume

	Change in paw volume (ml) and percent inhibition (%)										
Group	0 h	1 h	%	2 h	%	3 h	%	4 h	%	5 h	%
TE	0.43 ± 0.01	0.57 ± 0.01	–	0.73 ± 0.01	–	0.76 ± 0.01	–	0.81 ± 0.02	–	0.88 ± 0.04	–
ASA	0.42 ± 0.01	0.51 ± 0.02^a3c1d1^	36.1	0.53 ± 0.01^a3c3d3g3^	63.9	0.58 ± 0.02^a3c3d3h1^	50.8	0.64 ± 0.01^a3c3d3g3^	42.6	0.67 ± 0.01^a3c3d3g3^	45.3
CF 100	0.43 ± 0.01	0.57 ± 0.01	4.5	0.68 ± 0.01	15.1	0.72 ± 0.03	11.4	0.79 ± 0.01	4.8	0.85 ± 0.01	6.3
CF 200	0.44 ± 0.01	0.56 ± 0.01	7.7	0.67 ± 0.01	18.2	0.72 ± 0.02	12.9	0.78 ± 0.02	9.5	0.85 ± 0.01	9.2
CF 400	0.45 ± 0.01	0.58 ± 0.01	2.3	0.67 ± 0.01^a1h3^	24.4	0.70 ± 0.01^a1h1^	22.8	0.76 ± 0.01^a2f1h3^	18.0	0.80 ± 0.01^a3h3^	20.8
MF 100	0.42 ± 0.01	0.54 ± 0.01	11.3	0.63 ± 0.02^a3h1^	26.8	0.67 ± 0.01^a3b3^	24.0	0.71 ± 0.01^a3h1^	22.8	0.76 ± 0.01^a3h2^	23.4
MF 200	0.45 ± 0.01	0.57 ± 0.01	3.8	0.64 ± 0.01^a3h2^	33.0	0.69 ± 0.01^a2b3^	25.2	0.73 ± 0.01^a3h2^	25.4	0.78 ± 0.01^a3h3^	26.3
MF 400	0.44 ± 0.01	0.54 ± 0.02	21.8	0.58 ± 0.01^a3^	52.2	0.64 ± 0.02^a3b1^	37.2	0.68 ± 0.03^a3e3^	36.8	0.71 ± 0.01^a3^	39.7

Values are expressed as Mean ± S.E.M (*n* = 6); ^a^against the control, ^b^against the standard drug, ^c^against CF100, ^d^against CF200, ^e^against CF400, ^f^against MF 100, ^g^against MF 200, ^h^against MF 400. ^1^ *P* < 0.05, ^2^ *P* < 0.01, ^3^ *P* < 0.001; *TE* tween 80, *ASA*: aspirin, CF and MF refers to chloroform and methanol fractions of *Moringa stenopetala* respectively; control received 2% Tween 80, 10 ml/kg, whereas:ASA, standard received Aspirin 100 mg/kg orally; Numbers refer to dose in mg/kg

In distilled water treated group, the sub-plantar injection of carrageenan produced edema development which progressively increased with time. Compared to the control group, the administration of all doses of the aqueous fraction and that of standard drug showed statistically significant (*p* < 0.001) inhibitory effect on mean increase in paw volume starting from the second hour after carrageenan injection. Oral administration of AF (100, 200, 400 mg/kg) and aspirin maximally reduced the edema with 52.1%, 59.9%, 67.0% and 62.8% inhibition at 2 h (Table 6).

**Table 6:** Effect of the aqueous fraction of *Moringa stenopetala* on carrageenan- induced paw model and percent inhibition from increase in paw volume

	Change in paw volume (ml) and percent inhibition (%)										
Group	0 h	1 h	%	2 h	%	3 h	%	4 h	%	5 h	%
DW	0.43 ± 0.01	0.56 ± 0.01	–	0.71 ± 0.01	–	0.73 ± 0.01		0.80 ± 0.01	–	0.88 ± 0.02	–
ASA	0.42 ± 0.01	0.51 ± 0.02	35.6	0.53 ± 0.01^a3c1^	62.8	0.58 ± 0.02^a3^	48.1	0.64 ± 0.01^a3^	42.4	0.67 ± 0.01^a3^	45.8
AF 100	0.46 ± 0.02	0.55 ± 0.02	30.3	0.59 ± 0.01^a3e1^	52.1	0.62 ± 0.01^a3e1^	47.4	0.66 ± 0.02^a3^	45.6	0.72 ± 0.02^a3e1^	42.0
AF 200	0.44 ± 0.02	0.52 ± 0.01	34.8	0.55 ± 0.01^a3^	59.9	0.59 ± 0.01^a3^	51.3	0.63 ± 0.01^a3^	48.8	0.68 ± 0.01^a3^	46.9
AF 400	0.43 ± 0.02	0.50 ± 0.01^c1^	47.0	0.53 ± 0.01^a3c1^	67.0	0.56 ± 0.02^a3c1^	60.1	0.60 ± 0.02^a3b1^	54.9	0.65 ± 0.01^a3c1d1^	51.8

Values are expressed as Mean ± S.E.M (*n* = 6); ^a^against the control, ^b^against the standard drug, ^c^against AF100, ^d^against AF200, ^e^against AF400, ^1^ *P* < 0.05, ^2^ *P* < 0.01, ^3^ *P* < 0.001; *DW* distilled water, *ASA* aspirin, AF refers to aqueous fraction of *Moringa stenopetala*

## Discussion

The present study was aimed to evaluate the analgesic and anti- inflammatory activity of the solvent fractions of *Moringa stenopetala* in mice models of pain and inflammation. *M. stenopetala* has a popular reputation in Ethiopian folk medicine for the treatment of different ailments including pain [22]. Moreover, its in-vivo analgesic and anti-inflammatory activity was reported recently [25]. Therefore this study attempted to further evaluate the analgesic and anti-inflammatory activities of the solvent fractions of the plant.

In the acute toxicity study, none of the animals showed behavioral, neurological or physical changes characterized by symptoms such as reduced motor activity, restlessness, convulsions, coma, diarrhea and lacrimation at the limit dose of 2000 mg/kg of the solvent fractions of *M. stenopetala*. Moreover, no mortality was observed in 24 h as well as in the next 14 days. Thus, The LD_50_ of all the three fractional extracts of the plant was estimated to be above 2000 mg/kg. The result from acute toxicity study of the different doses of three solvent of *M. stenopetala* indicated that no lethality observed within 24 h and there are no behavioral changes within 14 days. The finding is inline with a study done by Musa et al. [24] for the butanol fraction of *M. stenopetala.* According to WHO hazard classification, all of the three fractions with LD_50_ > 2000 mg/kg can be designated as “unlikely to be hazard” [38]. So we can conclude that the fractions are safe for further study.

In all analgesic and anti-inflammatory models, male mice were used. Because it is suggested that experimental pain sensitivity changes across the menstrual cycle [39] and on the fact that estrogen exerts anti-inflammatory activity [40]. So as to avoid any fluctuation in the results female mice were excluded.

Several studies have validated the use of analgesic and anti-inflammatory medicinal plants by investigating the biological activity of extracts/fractions of plants. In this study the radiant tail flick test, a thermal method [41], to investigate central analgesic activity [42]and acetic acid-induced writhing response, a chemical method [43], to observe its peripheral analgesic effects were used.

In radiant tail flick test, application of thermal radiation to the tail of mouse provokes tail withdrawal with a vigorous movement, called tail flick. Tail flick test is an objective and quantifiable measure of pain that has been used for assessing anti-nociceptive activity of various drugs given systemically in both rats and mice. This test can involve both spinal and supra spinal structures, depending on the intensity of the radiant heat stimulation [44], The method is one of the most common tests of nociception that is based on a phasic stimulus of high intensity pain. The method was selected to investigate central antinociceptive activity because it had several advantages, particularly the sensitivity to strong antinociceptives and limited tissue damage [45].

In this study, a 10 s cut-off time was appointed for prevention of tissue damage on mice. The lengthening of the pretreatment latency time was exhibited after the administration of *M. stenopetala* fractions which is related to an analgesic action of the plant. The effectiveness of analgesic agents in the tail-flick pain model is highly correlated with relief of human pain perception [14]. This might strongly confirm the traditional claim of the plant. In this test, the highest dose of chloroform and all doses of methanol and aqueous fraction have shown a significant analgesia in comparison with the control (*p* < 0.01). Similarly, aqueous extract at doses of 200 and 400 mg/kg significantly increased the pain threshold which is even comparable to that of morphine at 90 and 120 min. The observed central analgesic effect of the fractions might be mediated by enhancing the release of endogenous peptides such as endorphin and enkephalin from the periaqueductal grey matter (PAG) [25] or the phytochemical constituents particularly found in the aqueous fraction might have pharmacological effect of opioid receptor activation.

In the second analgesic model, the acetic acid induced writhing test, also commonly known as abdominal contraction test, is used for a reliable and rapid evaluation of peripheral analgesic action of the plant. The test has long been used as a screening tool to evaluate antinociceptive and anti-inflammatory properties of new substances [46]. Pain sensation in this writhing method elicited by acetic acid is believed to act indirectly by inducing the release of prostaglandins as well as lipooxygenase products into the peritoneum which stimulate the nociceptive neurons on the sensory nerve fibers [47]. Acetic acid induced writhing test is a model of visceral pain. It is very sensitive and able to detect antinociceptive effects of compounds at dose levels that may appear inactive in other methods like the tail-flick test.

Unlike the previous method, only the higher dose of chloroform have shown a reduction in the writhing response (*p* < 0.01), the lowest two doses did not exhibited significant protections from pain sensation, it might be because of low concentration of secondary metabolites in the two doses, and this argument is further supported with the fact that the fractions were found to be dose dependent (R^2^ = 0.9985 for CF, R^2^ = 0.9992 for MF and R^2^ = 0.9893 for AF). In all administered doses of the methanol and aqueous fractions, there were significant (*p* < 0.001) reduction of pain sensation. The results further showed their analgesic effect was comparable with aspirin, which indicates the presence phytoconstituents in the fractions that possess analgesic activity with increasing dose. Similar to the tail flick test, the aqueous fraction at the highest dose (400 mg/kg), showed the maximal protection (58.0%). Therefore, from the result we strongly suggest that the pharmacological mechanism for the analgesic action of the plant may be somewhat linked reduction of prostaglandin synthesis due to their inhibitory role in lipooxygenase and/or cyclooxygenase pathway [35].

The analgesic action of the plant might be attributed to its phytochemical constituents. Reports showed that flavonoids, terpenoid and steroids which are found in AF,MF and CF of the plant, respectively, has inhibitory role in the production of prostaglandins [48] and free radical scavenging activity of flavonoids [35]. Alkaloids which are the pythocchemicals presented in methanol and chloroform fractions, also showed to exert their analgesic effect by interfering with neurotransmitter that enhance pain sensation in the CNS [49].

To evaluate the anti-inflammatory effect of the plant, carrageenan induced paw edema that is a widely used model to determine an anti-inflammatory activity of drugs as well as to study the mechanisms involved in inflammation was used. Since it is associated with several mediators, the model is a suitable in-vivo model to study anti-inflammatory effects of natural products [50]. Three phases represents the occurrence of carrageenan induced edema which is linked to the mediators. The initial phase is attributed to the action of mediators such as histamine and serotonin between 0 and 1.5 h post-carrageenan injection while the second phase (1.5–2.5 h) is contributed by bradykinin. In the third phase (2.5–6 h) prostaglandins play a major role in the development of inflammatory reaction [51].

In the present study, acute inflammation was produced by an injection of carrageenan in the right hind paw of the mice [52]. During injection, inflammatory response produced increase in vascular permeability and cellular infiltration leading to edema formation (an increase of paw volume), as a result of extravasation response in mice. The MF and AF had shown a significant (*p* < 0.001) inhibition from an increase in paw edema starting from the second hour after carrageenan injection. Since the methanol and aqueous fractions had reduced from paw edema during the late phases (2 h after carrageenan injection), it is possible that the fractions could have inhibitory effect on mediators such as prostaglandins, bradykinin and leukotriens or it could have inhibited the synthesis or release of these mediators and/or had free radical scavenging activity [53]. The highest anti-inflammatory activity of the extract was seen in the aqueous fraction in comparison to the standard drug. These facts may collectively indicate that the methanol and aqueous fractions of *M. stenopetala* may exert their action also by inhibiting COX, free radical scavenging activity and inhibiting subsequent prostaglandin synthesis. The anti-inflammatory activity of many plants is also related to the presence of saponins [35], terpenoids [54], alkaloids [55], glycosides [56] and tannins [35]. Thus, it can be said that the anti-inflammatory action of the extract observed in carrageenan induced paw edema model could possibly be due to the presence of alkaloids, glycosides, flavonoids, saponins and terpenoids. Generally the differences in the analgesic and anti-inflammatory effect of chloroform, methanol and aqueous fractions might be, at least in part, due to variability in composition, concentration and activity of those active principles detected in phytochemical screening tests, as these solvents differ in polarity index.

## Conclusion

The pharmacological tests performed in the present study confirmed the analgesic and anti-inflammatory activity of the three fractions of the leaves extracts of *M. stenopetala* and revealed that possessed a varying degree of central, peripheral analgesic and acute anti-inflammatory activity that could be attributed to the presence of bioactive agents including flavonoids, tannins, terpenoids, saponins, steroids, glycosides and alkaloids that might have acted separately or synergistically. Anti-nociceptive and anti-inflammatory properties of the fractions are probably mediated via inhibition of prostaglandin synthesis as well as central inhibitory mechanisms. The data collectively indicate that the aqueous fraction being the most active fraction followed by the methanol fraction and then chloroform fraction in all the three models. The results from the present study suggest that chemical constitute of the plant could serve as a lead compound in the development of new analgesic and anti-inflammatory agent.

## Abbreviations

AF: Aqueous fraction
CF: Chloroform fraction
MF: Methanol fraction
MO: Morphine

## References

[1] IASP ,International Association for the Study of Pain: Pain Definitions” available on http://www.iasp-pain.org/taxonomy, 2015. Retrieved 26 November 2015.

[2] Rajagopal M (2006). Pain - Basic considerations. Indian J Anaesth.

[3] Debon D, Hoeksema L, Hobbs R (2013). Caring for Patients with Chronic Pain: Pearls and Pitfalls. J Am Osteopath Assoc.

[4] Breivik H, Eisenberg E, O’Brien T (2013). The indivvidual and societal burden of chronic pain in Europe: The case for strategic prioritization and action to improve knowledge and availability of appropriate care. BMC Public Health.

[5] Underwood J (2004). General and Systematic Pathology.

[6] Kumar R, Clermont G, Vodovotz Y (2004). The dynamics of acute inflammation. J Theor Biol.

[7] Shojaii A, Motaghinejad M, Norouzi S, Motevalian M (2015). Evaluation of Anti -inflammatory and Analgesic Activity of the Extract and Fractions of *Astragalus hamosus* in Animal Models. Iran J Pharm Res.

[8] Barnes P (2006). How corticosteroids control inflammation: Quintiles Prize Lecture 2005. Br J Pharmacol.

[9] Nash R, Yates P, Edwards H (1999). Pain and administration of analgesia: what nurses say?. J Clin Nurs.

[10] Köksal M, Gökhan N, Küpeli E et al. Analgesic and Antiinflammatory Activities of Some New Mannich Bases of 5-Nitro-2-Benzoxazolinones. Arch Pharm Res 2007*;* 30(4): 419-424. 10.1007/BF02980214 17489356

[11] Munir F, Yarker J, Haslam C (2007). Use of prescribed medication at work in employees with chronic illness. Occup Med.

[12] Niemi T, Taxell C, Rosenberg P (1997). Comparison of the effect of intravenous ketoprofen, ketorolac and diclofenac on platelet function in volunteers. Acta Anaesthesiol Scand.

[13] Mital R, Edwin C, Suresh B (2015). Investigation of centrally and peripherally acting analgesic and anti-inflammatory activity of biological immune response modulator (an Amazonian plant extract) in animal models of pain and inflammation. Int J Basic Clin Pharmacol.

[14] Vittarao A, Shanbhag T, Kumari K (2011). Evaluation of anti-inflammatory and analgesic of alcoholic extract of Kaemperia Galagna in rats. Indian J Physiol Pharmacol.

[15] Bekele E: Study on Actual Situation of Medicinal Plants in Ethiopia. Prepared for Japan association for international Collaboration of Agriculture and Forestry, 2007; Addis Ababa.

[16] Mesfin K, Tekle G, Tesfay T (2013). Ethnobotanical study of traditional medicinal plants used by indigenous people of Gemad District, Northern Ethiopia. J Med Plants Stud.

[17] Giday M (2001). An ethnobotanical study of medicinal plants used by the Zay people in Ethiopia. CBMs Skritserie.

[18] Yirga G (2010). Ethnobotanical study of medicinal plants in and around Alamata, Southern Tigray, Northern Ethiopia. Curr Res J Biol Sci.

[19] Bekele G, Reddy P (2015). Ethnobotanical study of medicinal plants used to treat human ailments by Guji Oromo tribes in Abaya District, Borana, Oromia, Ethiopia. Univ J Plant Sci.

[20] Teklehaymanot T, Giday M (2010). Ethnobotanical study of wild edible plants of Kara and Kwego semi-pastoralist people in Lower Omo River Valley, Debub Omo Zone, SNNPR, Ethiopia. J Ethno Biol Ethno Med.

[21] Jiru D, Sonder K, Alemayehu L, Mekonen Y, and Anjulo A. Leaf yield and Nutritive value of *Moringa stenopetala* and *Moringa oleifera* Accessions: Its potential role in food security in constrained dry farming agroforestry system. Moringa and other highly nutritious plant resources: Strategies, standards and markets for a better impact on nutrition in Africa. Accra, Ghana; 2006: p. 1–14.

[22] Ghebreselassie D, Mekonnen Y, Gebru G, Ergete W, Huruy K (2011). The effects of Moringa stenopetala on blood parameters and histopathology of liver and kidney in mice. Ethiop J Health Dev.

[23] Mekonnen Y, Amare G, Ermias D, et al. The multi-purpose Moringa tree: Ethiopia Institute of Pathobiology, Addis Ababa University, Addis Ababa, Ethiopia. 1996;10:111-118.

[24] Musa A, Vata P, Debella A (2015). Acute toxicity studies of butanol fraction of leaves of *Moringa stenopetala* in rats. Asian Pac J Health Sci.

[25] Geremew H, Shibeshi W, Engidawork E, Tamiru W (2015). Evaluation of Analgesic and Anti-inflammatory activity of *Moringa stenopetala* Bak. (Moringaceae) in mice. Ethiop J Pharmaceut.

[26] Negus S, Vanderah TW, Brandt MR, Bilsky EJ, Becerra L, Borsook D (2006). Preclinical assessment of candidate analgesic drugs: recent advances and future challenges. J Pharmacol Exp Ther.

[27] Institute for Laboratory Animal Research (ILAR): Guide for the care and use of laboratory animals. Washington DC: National Academy Press. 1996.

[28] Ayoola G, Coker H, Adesegun S, Adepoju-Bello A, Obaweya K, Ezennia E, Atangbayila T (2008). Phytochemical screening and antioxidant activities of some selected medicinal Plants used for malaria therapy in Southwestern Nigeria. Trop J Pharm Res.

[29] Sasidharan S, ChenY SD (2011). Extraction, isolation and characterization of bioactive compounds from plants’ extracts. Afr J Tradit Complement Altern Med.

[30] OECD: Guidelines for Testing of Chemicals: Guideline 425: Acute Oral Toxicity. Paris, France. The Organization of Economic Co-operation and Development.2008.

[31] D'amour F, Smith D (1941). A method for determining loss of pain sensation. J Pharmacol Exp Ther.

[32] Milind P, Monu Y (2013). Laboratory models for screening analgesics. Int Res J Pharm.

[33] Torres IL, Vasconcellos AP, Cucco SNS (2001). Effect of repeated stress on novelity induced anti-nociception in rats. Braz J Med Biol Res.

[34] Koster R, Anderson M, De Beer E (1959). Acetic acid analgesic screening. Fed Proc.

[35] Hernández-Ortega M, Ortiz-Moreno A, Hernández-Navarro M, Chamorro-Cevallos G, Dorantes-Alvarez L, et al. Antioxidant, antinociceptive and anti-inflammatory effects of Carotenoids extracted from dried pepper (*Capsicum annuum* L). J Biomed Biotechnol. 2012:1-10. 10.1155/2012/524019 PMC3468166 23091348

[36] Winter C, Risley E, And Nuss C (1962). Carrageenan-induced oedema in the hind paw of the rat as an assay for anti-inflammatory drugs. Proc Soc Exp Biol Med.

[37] Olukunle JO, Adenubi OT, Oladele GM (2011). Studies on the anti-inflammatory and analgesic properties of *Jatropha curcas* leaf extract. Acta Vet Brno.

[38] WHO. Hazard classification-acute LD50 values of formulated products. In: The guidebook to the registration of public health pesticides and repellents against Vectors.1975.

[39] Bartley EJ, Fillingim RB (2013). Sex differences in pain: A brief review of clinical and experimental findings. Br J Anaesth.

[40] Vegeto E, Benedusi V, Maggi A (2008). Estrogen anti-inflammatory activity in brain: A therapeutic opportunity for menopause and neurodegenerative diseases. Front Neuroendocrinol.

[41] Siddalingappa C, Rajesh T, Kudagi B (2012). Evaluation of analgesic and anti-inflammatory activites of Tinospora cordifolia in rodents. Int J Basic Med Sci.

[42] Silva J, Abebe W, Sonsa SM (2003). Analgesic and antiinflammatory effects of essential oil of Eucalyptus. J Ethnopharmacol.

[43] Marzouk B, Marzouk Z, Fenina N (2011). Anti-inflammatory and analgesic activities of Tunisian Citrullus colocynthis Schrad, Immature fruit and seed organic extracts. Eur Rev Med Pharmacol Sci.

[44] Le Bars D, Gozariu M, Cadden SW (2001). Animal models of nociception. Pharmacol Rev.

[45] Mandegary A, Sayyah M, Heidari MR (2004). Antinociceptive and anti-inflammatory activity of the seed and root extracts of *Ferula gummosa Boiss* in mice and rats. Daru.

[46] Collier H, Dinneen L, Johnson C, Schneide C (1868). Abdominal constriction response and its suppression by analgesic drugs in mouse. Br J Pharmacol.

[47] Satyanarayana P, Jain N, Singh S, Kulkarni S (2004). Effect of selective inhibition of cyclooxygenase-2 on lipopolysaccharide induced hyperalgesia. Inflammopharmacol.

[48] Awad A, Toczek J, Fink C (2004). Phytosterols decrease prostaglandin release in cultured P388D1/MAB macrophages. Prostag Leukot Essent Fatty Acids.

[49] Reanmongkol W, Subhadhirasakul S, Thienmontree S, Thanyapanit K, Kalnaowakul J, Sengsui S (2005). Anti-nociceptive activity of the alkaloid extracts from *Kopsia macrophylla* leaves in mice. J Sci Technol.

[50] Woldesellassie M, Eyasu M, Kelbessa U (2011). In vivo anti-inflammatory activities of leaf extracts of Ocimum lamiifolium in mice model. J Ethnopharmacol.

[51] Carey W, Rao V, Kumar R, Mohan K (2010). Anti-inflammatory and analgesic activities of methanolic extract of *Kigelia pinnata* DC flower. J Ethnopharmacol.

[52] Juhas S, Bujnakova D, Rehak P, Ciko S, Czikkova S, Vesela J, Iikova G, Koppel J (2008). Anti-Inflammatory effects of thyme essential oil in mice. Acta Vet Brno.

[53] Yang R, Tsou S, Lee T, Wang M, Sang S, Hwang LS, Ho CT (2006). Moringa: a Novel Plant Rich in Antioxidants, Bioavailable Iron, and Nutrients. Herbs: Challenges in Chemistry and Biology.

[54] Çadirci E, Suleyman H, Gurbuz P, Kuruuzum A, Güvenalp Z, Demirezer L. Anti-infl ammatory effects of different extracts from three *Salvia* species. Turk J Biol. 36:59–64.

[55] Souto A, Tavares J, da Silva M (2011). Anti-Inflammatory Activity of Alkaloids: An Update from 2000 to 2010. Molecules.

[56] Hosseinzadeh H, Younesi H (2002). Antinociceptive and anti-inflammatory effects of *Crocus sativus* L. stigma and petal extracts in mice. BMC Pharmacol.

